# Supplementary material for: Socio-cultural and structural barriers influencing parents’ knowledge and access to information on schistosomiasis in children around Ugandan Lakes
Source: PLoS Negl Trop Dis. 2025 May 8;19(5):e0013050. doi: 10.1371/journal.pntd.0013050 (PMC12091885; doi:10.1371/journal.pntd.0013050)
Supplement: S1 Text — (DOCX) [file pntd.0013050.s001.docx]

## TOOL 5: Interview guide addressed to

## parents/guardians of children 1-5 years old

1. **Background information**

| ***No*** | ***Background information*** | ***Response*** |
| --- | --- | --- |
|  | Participant ID Number |  |
|  | Interviewer’s code or name |  |
|  | District, sub-county, village |  |
|  | Sex |  |
|  | Age |  |
|  | Highest education achieved |  |
|  | Occupation |  |
|  | Date of interview  Start time of interview  End time of interview |  |

***Suggested ice-breaker:***

*(But feel free to choose a subject that feels right to you)*
What are some of the challenges encountered in the community you live in these days?

*Probe on the following to generate discussion if needed: Health, education, climate, water, poverty, unemployment, etc.*

**1. Knowledge and perception of bilharzia and practices**

1.1 What is this disease called here? What does the name mean in your language, and do you know why it is called that way? Are there any other names for bilharzia?

1.2 What do you think of when you hear about bilharzia?

1.3 Is it an issue in your community? If so, how? In which way? If not, why isn’t it?

1.4 What are the signs that a person has bilharzia in your community?

1.5 Are the signs different in children? If so, what are they?

1.6 How do people get infected with bilharzia? *(Pathways of transmission)*

1.7 What are effective ways to prevent being infected with bilharzia?

1.8 What do you and your community do to prevent getting a bilharzia infection (if any)?

1.9 Do you and your family face any challenges in your daily lives concerning the prevention and the risk of infection? If yes, which challenges? *Probe on the following (if not mentioned during the discussion and ask why)*

- *Contact to potentially contaminated water during household and family activities (e.g. bathing, laundry, collecting water, washing dishes, swimming of children) or occupational activities (e.g. fishermen, water sales men, laundry women/“dobbies”, livestock)?*
- *Is safe water available? If yes, is it affordable?*
- *Are latrines available, and used?*

**2. Uptake of campaign treatments and health-seeking behaviour**

2.1 What do people in your community do if they know they are infected with bilharzia?

2.2 What do people do to treat bilharzia? *(For the interviewer: Ask for examples, and get a sense of what kind of treatment they turn to for bilharzia, especially traditional medicine, western medicine, religion, or others.)*

2.3 What about treatment specifically for children 1-5 years old. Do people in your community have a way of treating young infected children? Please explain if so, what it is. How well does it work? What are the positives and negatives, if any, of such treatment?

2.4 Which experience do you have in general with treatments for other illnesses for children 1-5 years old? Were they positive or negative experiences and why?

2.5 What do you think of Mass Drug Administrations in general? For adults? For children?

2.6 In your opinion, who mostly influences the decision-making processes regarding child health in families of your community?

**3. Potential pediatric treatment of bilharzia**

3.1 A new drug is being developed that treats bilharzia in children between 1 and 5 years old. The new pill is smaller than the adult version, tastes better, can be dissolved in water, and has just the right dose for young children. As a parent, what do you think about this news?

3.2 Using this child-friendly formulation, the Ugandan Ministry of Health is planning to conduct a treatment programme in the form of a mass drug administration (MDA) for children aged 1-5 years for bilharzia. Would you want your child to take part in this treatment programme? Please explain why or why not.

3.3 Let’s imagine that before administering the drug, all children have the possibility of having their **urine or stool tested** for bilharzia. Do you think that having diagnostic testing available would influence your decision as to whether your child should participate in mass treatment for bilharzia? Why or why not? Please explain.

3.4 In your view, what could be the appropriate methods for drug distribution for this new drug formulation? Who should be involved and how should it be done? Please explain your thoughts. *Probe on the following:*

- *Door-to-door by CHVs/VHTs*
- *At a fixed point, e.g. health facilities, schools/ECDs, religious institutions, marketplaces, etc.*
- *Child Health Day*
- *Other programmes or platforms*
- *Combination of all the above platforms – mixed approach, etc.*

*🡪 Why do you think these methods may or may not work in your community?*

**4. Communication about bilharzia treatment**

4.1 Which sources of information do you trust related to health care and in particular drug treatment programmes? Why do you trust them?

4.2 What do you think about public health campaigns? Have you had positive or negative experiences with them? *Ask for examples. If none are mentioned, offer the following examples to prompt their opinions: COVID-19 and family planning.*

4.3 How do you usually hear about or receive information related to the mass treatment programmes (mass drug administrations / MDA) that take place in your community?

4.4 What means of communication would you recommend for a mass treatment programme in order to adequately inform parents of children aged 1-5 years in this community? Why?
*Probe on the following (if not mentioned during the discussion):*

- *Print media: Posters, brochures, flyrs, banners, newspapers*
- *Broadcast media: Radio or television (which station?)*
- *(Mobile) Phone: Hotline or text messages*
- *Social media like Facebook, Instagram, Twitter, tik tok*
- *Community-based: Community leaders, community groups, community meetings*
- *Health facilities: Healthcare Providers or CHVs/VHTs*
- *At other institutions: Religious institutions (churches, mosques, other), schools*
- *Public events: Roadshows, theatre-based public health education, public debates*
- *Personal communication: Door-to-door sessions by mobilizers to discuss in privacy, family members, and other persons like neighbours, friends, shop owners*

4.5 What aspects of the MDA would be important to emphasise so that all parents of young children are able to make an informed and responsible decision about their participation? *Probe on the following (if not mentioned during the discussion):*

- *Benefits of treatment for the individual child*
- *The reason for treating all children whether infected or not*
- *The reason for now treating children 1-5 when previously children were only included in MDAs from age 6 on*
- *Benefits of the treatment for the community (decreases the spread of disease)*
- *Sensitization about potential side effects*
- *Measures to take if side effects occur*
- *That they are free to take part or not; the choice is theirs*

4.6 Some parents may be sceptical about mass drug administration, especially when their small children are supposed to participate. In your opinion, what steps should be taken to ensure that these parents are able to make an informed and responsible choice regarding the administration of the drug to their children?

**5. Experiences with Praziquantel**

- 1. We are interested in your thoughts about any negative consequences of taking the existing anti-bilharzia medication (Praziquantel). In your opinion, are there any? If so, what are they?
  2. From what you know, can there be side effects from taking Praziquantel? If so, which? If so, do the side effects bother you? Tell us about it.
  3. In your experience, is information about potential side effects adequately provided for people taking the medication? Who provides this information? (E.g: health workers, CHVs, friends and family, social media)
  4. Is healthcare available and affordable for those who might experience side effects of Praziquantel?
  5. If you or your child experienced side effects, would you report (or: inform about) them to anyone? If so, to whom, and why? *(For the interviewer: with this question, we are interested in the monitoring and surveillance of side effects, such as reporting to authorities like teachers, health workers, CHVs, etc.)*
  6. In your opinion, what measures should be taken to address possible side effects of the new child-friendly drug distributed in MDAs? *If not mentioned ask: which information would you like to receive concerning potential side effects?*

**6. Suggestions**

6.1 What else should be added to help improve the administration of this drug to the young child/children in your care?

6.2 Do you have any other thoughts or ideas about Bilharzia treatment or prevention that you would like to share with us?

**Thank you for your participation.**
