# Supplementary material for: Socio-cultural and structural barriers influencing parents’ knowledge and access to information on schistosomiasis in children around Ugandan Lakes
Source: PLoS Negl Trop Dis. 2025 May 8;19(5):e0013050. doi: 10.1371/journal.pntd.0013050 (PMC12091885; doi:10.1371/journal.pntd.0013050)
Supplement: Attachment — Submitted filename: SUGGESTION_REVIEW.pdf [file pntd.0013050.s002.pdf]

"Socio-cultural and structural barriers influencing parents' knowledge and access to information on schistosomiasis in children around Ugandan Lakes" (PNTD-D-24-00908) for PLOS Neglected Tropical Diseases.

Dear authors,

I would like to congratulate you on the excellent work done in this study. The detailed methodology and analysis offer significant contributions to understanding the socio-cultural and structural barriers to accessing information about schistosomiasis. The research will undoubtedly have a positive impact on public health policies and the fight against the disease. I appreciate the opportunity to review this work. Therefore, I would like to bring your attention to some general points outlined below:

These considerations can help ensure that the methodology is robust and that the study's conclusions are well-founded.

1. **Clarity in the Description of Methods**
   - Suggestion: The description of the methodology should be clear and detailed, especially regarding the selection of participants and the justification for using qualitative methods. Consider including more details on how the interviews and group discussions were conducted, such as the average duration of the interviews and the specific conditions in which they were conducted.
2. **Sampling and Representativeness**
   - Suggestion: Although purposive sampling is appropriate for qualitative studies, one might question whether the sample of 142 individuals, equally divided between the two districts, is sufficiently representative to capture the diversity of perceptions in different social and cultural contexts. I suggest that the authors justify the choice of sample size concerning the study's objectives.
3. **Potential Bias in Data Collection**
   - Suggestion: Comment on potential biases in data collection, such as the influence of Village Health Teams (VHTs) in recruiting participants or how the presence of translators might have affected the quality of the information collected. I recommend that the authors discuss the measures taken to mitigate these biases.
4. **Data Triangulation**
   - Suggestion: It is positive that the study used different data collection methods (interviews, focus groups, direct observation). However, it might be useful to discuss how data from different sources were

triangulated to strengthen the validity of the results. I suggest that the authors describe more explicitly how triangulation was performed.

5. **Data Analysis Approach**
   - Suggestion: The methodology mentions the use of an inductive and deductive thematic approach. As a reviewer, I suggest providing a more detailed explanation of how these approaches were integrated during data analysis and how thematic categories were developed and refined.
6. **Ethical Considerations**
   - Suggestion: Although ethical approval was obtained, it might be relevant for the authors to include more information on how confidentiality and informed consent were ensured in practice, especially in contexts where there is a high rate of illiteracy and language barriers.
7. **Methodological Limitations**
   - Suggestion: The authors should discuss more clearly the methodological limitations of the study, such as the impact of language barriers and high population mobility on data collection and interpretation of results.
8. **Generalization of Results**
   - Suggestion: Is there a possibility of generalizing the results to other regions or populations? It might be important for the authors to discuss the limitations regarding the generalization of the findings, given the cultural and contextual specificities of the two districts studied.

Results Analysis

This analysis highlights the main findings of the study and suggests areas for discussion and improvement, helping to strengthen the contribution of the article to the field of public health.

1. **Awareness and Knowledge about Schistosomiasis**
   - Comment: The results reveal a significant level of awareness about schistosomiasis among parents, which is encouraging. However, there are important gaps in knowledge regarding the transmission and prevention of the disease. The identification of erroneous beliefs, such as the association between schistosomiasis and

witchcraft, underscores the need for more focused and culturally sensitive educational campaigns. I recommend that the authors discuss how these beliefs can be effectively addressed in future interventions.

2. **Information Sources and Trust in VHTs**
   - Comment: The identification of Village Health Teams (VHTs) as a primary source of information is a relevant finding. However, the limited training of VHTs regarding schistosomiasis suggests that they may not be fully equipped to provide accurate information. I suggest that the authors consider discussing strategies to improve the training of VHTs so that they can play a more effective role in disseminating health information.
3. **Socio-Cultural and Structural Barriers**
   - Comment: The analysis of barriers, such as high population mobility and illiteracy, provides valuable insight into the challenges faced in communicating health information. The identification of language barriers and limited trust in written information sources are critical issues that need to be addressed to ensure more effective information dissemination. I suggest that the authors discuss possible approaches to overcoming these barriers, such as using visual communication or oral narratives.
4. **Information Dissemination and Campaign Impact**
   - Comment: The results indicate that the dissemination of information about schistosomiasis has been fragmented and, in many cases, limited to logistical issues. The lack of comprehensive informational campaigns may be contributing to the persistence of knowledge gaps among parents. It would be helpful if the authors discussed the need for more structured and continuous health campaigns, especially those focused on hard-to-reach areas.
5. **Acceptance and Use of Praziquantel**
   - Comment: The acceptance of praziquantel as an effective treatment method is positive, but reports of side effects and the lack of information on how to deal with them are concerning. I recommend that the authors consider discussing the importance of providing complete information about side effects during MDA campaigns, which could improve acceptance and adherence to treatment.
6. **Suggestions for Future Actions**

- Comment: Based on the results, future actions could include developing training programs for VHTs, creating more inclusive health campaigns, and implementing communication strategies that take into account the identified socio-cultural and structural barriers. I recommend that the authors consider exploring these suggestions in more detail, offering specific recommendations for future interventions.
