# Supplementary material for: Socio-cultural and structural barriers influencing parents’ knowledge and access to information on schistosomiasis in children around Ugandan Lakes
Source: PLoS Negl Trop Dis. 2025 May 8;19(5):e0013050. doi: 10.1371/journal.pntd.0013050 (PMC12091885; doi:10.1371/journal.pntd.0013050)
Supplement: Attachment — Submitted filename: General Comments.pdf [file pntd.0013050.s004.pdf]

General Comments

The manuscript addresses an important topic related to socio-cultural and structural barriers impacting knowledge and access to information about schistosomiasis in children in Uganda. The study is highly relevant for advancing public health policies in endemic areas and integrating pediatric praziquantel formulations. The qualitative research provides valuable insights, but there are areas where clarity, validity, and applicability can be enhanced to strengthen the manuscript.

Strengths

1. **Scientific and Social Relevance**:
   - ✓ The topic is crucial for public health, addressing structural and cultural barriers that limit the effectiveness of biomedical interventions. The practical application of the findings is well-articulated.
2. **Qualitative Methodology**:
   - ✓ The use of interviews and focus groups was appropriate for exploring local perceptions. Employing both inductive and deductive analytical approaches adds credibility to the findings.
3. **Practical Recommendations**:
   - ✓ Suggestions for culturally tailored campaigns and training for Village Health Teams (VHTs) are concrete and actionable.

Constructive Criticisms and Suggestions for Improvement

1. Methodological Description

- **Observation**: The methodology section lacks detail about participant selection and criteria for data saturation.
- **Suggestion**: Provide a clear explanation of how participants were chosen and include a flowchart to illustrate the process.

2. Connection Between Findings and Solutions

- **Observation**: Local beliefs, such as perceptions of "kidaada," are mentioned but not fully integrated into the proposed strategies.
- **Suggestion**: Expand the discussion on how these beliefs could be addressed in health campaigns, possibly through cultural mediators or community dialogues.

3. VHT Capacity Building

- **Observation**: While VHTs are identified as key agents, their limitations (e.g., limited knowledge and education) are not sufficiently explored.

- **Suggestion**: Discuss specific training strategies for VHTs, including modules on health communication and cultural understanding.

4. Complementary Results

- **Observation**: The study focuses on qualitative findings, but additional quantitative data could enrich the context, such as adherence rates to MDA campaigns or local disease prevalence.
- **Suggestion**: Incorporate or reference relevant quantitative data to better contextualize the findings.

5. Structural Barriers

- **Observation**: High population mobility and language barriers are mentioned but lack practical solutions.
- **Suggestion**: Propose the use of community radio, visual materials, or audio-based resources to expand the reach of health messages.

6. Conclusion and Impact

- **Observation**: The conclusion could more clearly emphasize the potential impact of the findings on public policies and local health strategies.
- **Suggestion**: Include a paragraph on how the findings can inform interventions in other endemic contexts across sub-Saharan Africa.

Technical and Formatting Comments

1. **Abstract**:
    - ✓ Add numerical details (e.g., number of interviews and focus groups conducted) for greater clarity and alignment with PLOS NTDs standards.
2. **Title**:
    - ✓ The current title is clear and appropriate. However, a more concise version could be:
        - *“Barriers to Knowledge and Information on Pediatric Schistosomiasis in Ugandan Lakes”*.
3. **References**:
    - ✓ Ensure all in-text citations are correctly listed in the reference section.
4. **Tables and Figures**:
    - ✓ Verify that all tables and figures are formatted according to the journal's guidelines and include self-explanatory captions.
