# Supplementary material for: Socio-cultural and structural barriers influencing parents’ knowledge and access to information on schistosomiasis in children around Ugandan Lakes
Source: PLoS Negl Trop Dis. 2025 May 8;19(5):e0013050. doi: 10.1371/journal.pntd.0013050 (PMC12091885; doi:10.1371/journal.pntd.0013050)
Supplement: Attachment — Submitted filename: Letter_to_reviewers_05112024.docx [file pntd.0013050.s003.docx]

Ref: PNTD-D-24-00908

Manuscript title: Socio-cultural and structural barriers influencing parents’ knowledge and access to information on schistosomiasis in children around Ugandan Lakes

PLOS Neglected Tropical Diseases

Dear editors, dear reviewers,

Thank you for considering this manuscript for publication at PLOS Neglected Tropical Diseases. Thank you reviewer 1, 2 and 3 for taking the time to review this article and engage with the content and arguments. Your feedback has caused us to reflect on our presentation and make improvements to the manuscript based on your comments. Please see the table below for specific edits made.

Kind regards,

Lisa Sophie Reigl and co-authors

| **REVIEWERS’ COMMENTS** | **AUTHORS’ RESPONSES** |
| --- | --- |
| METHODS |  |
| **Reviewer #1:** The rationale for choosing villages within the two districts is not clear.  How large are the districts of Bugiri and Hoima? How much of the district populations reside in five selected villages in the Bugiri and the ten villages in Hoima?  How were these villages selected and what can be said about these villages in terms of the target population that the investigators have attempted to reach in this study?  Statistical concerns do not apply to a qualitative study in the same way they would to a quantitative study, but the question of who is included/excluded in the study population has bearing on how the research question is answered and the conclusions that can drawn. The process of selecting the study population should be outlined in more detail, with justification/rationale provided for the choices made. | Thank you for pointing this out. In section **2.2 Study area and setting**, we added the population numbers for the districts and selection criteria (underlined text).  “The study was conducted in two districts: Bugiri at Lake Victoria in southern Uganda, close to the Kenyan border, counting a population of 516,735 in 2014 (40), and Hoima at Lake Albert, western Uganda, close to the border with the Democratic Republic of Congo (DRC) with a projected population of 374,500 in 2020 (41). Through guidance from the Vector Control Division from the Ministry of Health, districts and sub-counties were selected that have a high schistosomiasis prevalence. The combined population of the chosen two sub-counties is approximately 34,000 with 7,500 households.”  Within this addition, we added two new references:  40. Republic of Uganda. Bugiri District. Local government n.d. [29.10.2024]. Available from: https://bugiri.go.ug/lg/location-size.  41. Republic of Uganda. Hoima District. Local government n.d. [29.10.2024]. Available from: https://hoima.go.ug/population/.  In **2.3 Study design, population and recruitment,** the underlined text was added:  “A purposive sampling technique was utilised to select both the villages and the participants for the study. One key selection criterion was that the villages had to be located near the lake shores. We chose interviewees who we believed could provide rich, detailed insights relevant to our research – focusing on individuals with pertinent experience or knowledge. Additionally, only those willing to participate were considered. With assistance from the local council officials or designated guides, our research team visited the households to conduct qualitative interviews.” |
| **Reviewer #1:** How did the in-depth interviews, key informant interviews and focus group discussions differ in terms of the data collection instrument? Were different questions asked? Or the same questions asked differently?  How did any differences get accounted for in the data analysis stage? Since there were different data collection modalities and different types of participants, some description of how those modalities differed in terms of both data collection and analysis is warranted. | The questions of the individual interview guides were formulated according to background of the targeted respondents.  The FGD guides were shorter, which means they contained fewer questions. Some questions were similar to the individual interview guides, however, FGD questions were formulated in a way as to generate dialogue and discussion amongst the participants, which then could be analysed accordingly. |
| **Reviewer #2:**   1. Clarity in the Description of Methods   Suggestion: The description of the methodology should be clear and detailed, especially regarding the selection of participants and the justification for using qualitative methods.  Consider including more details on how the interviews and group discussions were conducted, such as the average duration of the interviews and the specific conditions in which they were conducted.   2. Sampling and Representativeness    Suggestion: Although purposive sampling is appropriate for qualitative studies, one might question whether the sample of 142 individuals, equally divided between the two districts, is sufficiently representative to capture the diversity of perceptions in different social and cultural contexts. I suggest that the authors justify the choice of sample size concerning the study's objectives. 3. Potential Bias in Data Collection    Suggestion: Comment on potential biases in data collection, such as the influence of Village Health Teams (VHTs) in recruiting participants or how the presence of translators might have affected the quality of the information collected. I recommend that the authors discuss the measures taken to mitigate these biases. 4. Data Triangulation    Suggestion: It is positive that the study used different data collection methods (interviews, focus groups, direct observation). However, it might be useful to discuss how data from different sources were triangulated to strengthen the validity of the results. I suggest that the authors describe more explicitly how triangulation was performed. 5. Data Analysis Approach Suggestion: The methodology mentions the use of an inductive and deductive thematic approach. As a reviewer, I suggest providing a more detailed explanation of how these approaches were integrated during data analysis and how thematic categories were developed and refined. 6. Ethical Considerations   Suggestion: Although ethical approval was obtained, it might be relevant for the authors to include more information on how confidentiality and informed consent were ensured in practice, especially in contexts where there is a high rate of illiteracy and language barriers.   7. Methodological Limitations   Suggestion: The authors should discuss more clearly the methodological limitations of the study, such as the impact of language barriers and high population mobility on data collection and interpretation of results.   8. Generalization of Results   Suggestion: Is there a possibility of generalizing the results to other regions or populations? It might be important for the authors to discuss the limitations regarding the generalization of the findings, given the cultural and contextual specificities of the two districts studied. | Thank you for your suggestions on how to improve the methods section.   1. In **2.3 Study design, population and recruitment** the selection of participants is described:   “A purposive sampling technique was utilised to select participants for the study. The technique enabled us to select participants based on the groups targeted by the new paediatric praziquantel formulation, including its social mobilisation, sensitisation, and distribution (43). The selection of the parents/guardians (later only referred to as “parents”) was based on the age of their youngest child (below five years, PSAC), sex, and place of residence.”  We added the underlined sentence in **2.3. Study design, population and recruitment**:  “This study utilised a cross-sectional qualitative design to assess parents’ knowledge, preferred information sources and socio-cultural and structural barriers for communicating schistosomiasis information. This enabled researchers to identify and study various factors that potentially affect the implementation of paediatric treatment and to compare these findings across research sites.”  We added the underlined half-sentence on interview setting in **2.4 Data collection methods and instruments**:  “All interviews were conducted in private settings chosen by research participants, including homes, schools, health centres, private clinics, and offices.”  The following sentence was also added:  “The interviews resulted in 97h 24min of audio-recording, with an average time of 1h 13min for individual interviews and 1h 54min for FGDs.”   2. We added to **2.3 Study design, population and recruitment** the underlined sentence: “The sample size was determined by the participants’ profiles and data saturation.”   3. We added the underlined half-sentence and full sentence to the already named limitation in **4.5 Limitations of the study**:  “Depending solely on VHTs as guides to identify households with PSAC to participate in the study may have introduced a selection bias, e.g. that certain households could have been either intentionally or unintentionally overlooked, especially households that were not in good contact with VHTs. One parent was recruited independently from VHTs’ advice and was one of the few persons who said they did not take praziquantel themselves. At the same time, it was a thoroughly deliberated decision as VHTs are generally trusted locally and were, therefore, helpful in arranging the appointments.”   4. Data triangulation allowed us to learn about the perspectives of different actors involved in the experience of schistosomiasis illness, treatment and programming in the communities within which we worked. These different sources allowed us to lay out the perspectives in the results and came together in our analysis in the discussion section, which allowed for a deeper understanding of the factors that influenced individuals and different profile groups. The analysis process of coding data is described in the document and articulates that profile characteristics were taken into consideration during the analysis process. Please find further elaboration in section **2.5 Data analysis,** where we added now the underlined words:   “The researchers added memos and annotations to the software as they coded the data. After reading relevant codes, they were grouped into themes and analysed. Care was taken to identify patterns and differences between participant profiles, including personal demographic characteristics, district location and informant groups.”   5. Thank you for pointing this out. Indeed, we had mentioned this approach only in the abstract and missed to explain it in the method’s section. We added one sentence to section **2.5 Data analysis**:   “Topics and sub-themes, such as quality of information sources, the flooding in Hoima and the stricter fishing regulations iteratively arose during data collection, coding and further analysis, however reading scientific literature helped place the topics in relation to one another and review our data accordingly.”   6. The section **2.1 Ethical considerations** includes information on the informed consent. We added the underlined words:   “Ethical approval was obtained from the Ethics Committee at the Technical University of Munich (TUM) (731/21 S), by the Makerere University (MU) School of Social Sciences Research Ethics Committee (MAKSSREC 01.22.528), and by the Uganda National Council for Science and Technology (SS1269ES). All study participants took part voluntarily after receiving a study information sheet and being verbally informed about the rationale for the study, the study procedures, their rights as participants and being given the opportunity to ask for clarifications. Written informed consent was sought from each participant and an impartial witness in case of illiteracy of the participant.”   7. The topic “high population mobility” was given attention to in the results’ section **3.6.2 Information interruption due to population movements** and formed a key finding, but did not present a limitation to the study. Concerning the potential impact of language barriers, we added the underlined sentence to the paragraph in **4.5 Limitations of the study**:   “The presence of various languages among participants was addressed by employing local research assistants who each spoke several languages. However, most authors of this article depended on transcript translations. The co-authors from Uganda reviewed the analysis and interpretation accordingly.”   8. We added underlined half-sentence in **5. Conclusion**:   “Although the results of in-depth, qualitative research are not intended to be widely generalisable, our findings indicate the importance of considering contextual factors such as language barriers, level of literacy, population movements, rumours and trust in government programmes for planning the (pilot) roll-out of the schistosomiasis treatment for PSAC.” |
| **Reviewer #3:**  • In the methods (2.5), you state that transcripts were ‘where necessary, edited’ – please include a brief sentence on what they were edited for/in what way they were edited.  • What does s.b. stand for in Table 2? Can this be spelt out somewhere?  • Table 3 captures the key themes really clearly and succinctly. A great addition to the paper. Could you expand ‘Correctness of information provided dependent on knowledge of the parents’ information providers’ a little bit? | Thank you for your attention to details and your suggestions for improvement.  In **2.5 Data analysis**, we added the underlined sentence:  “Edits included, for example, correcting grammar to improve clarity and explaining locally used terms.”   - Change made in **Table 2:** s.b. => “somebody”**.** - **Table 3**: the mentioned sub-theme was changed and expanded now into:   “Quality of information provided dependent on knowledge of the parents’ information source: lower information quality provided by VHTs and community leaders, higher quality provided by teachers and health professionals.” |
| RESULTS ANALYSIS |  |
| **Reviewer #1:**  Please include a summary of the characteristics of the study villages (schistosomiasis burden; proximity to lake, school, market; community infrastructure observed) at the beginning of the results section.  A high-level description of the data (e.g., how much audio was recorded and how many pages of transcripts did that produce in the end?) would also be helpful so readers can appreciate the amount of enormous amount of time and effort that goes into both collecting and analyzing qualitative data.  In a study that is so focused on supporting the implementation of a treatment program in preschool-aged children, I was surprised that the authors buried their findings on parents knowledge of treatment so deep into their results section (it’s on the SIXTH page of the results!). The results seem to be structured in sort of a ‘kitchen sink’ way, where every topic covered in interviews and focus groups is addressed very briefly. This doesn’t allow the authors much room for elaboration or the inclusion of more than a quote or two per topic. It also makes it hard for the reader to extract a key message from the study’s findings. Instead of trying to fit so much into so little space, I would encourage the authors to lead with their top-line findings (e.g., what are the most important three points you would communicate to government partners?) and expand on those in the results and discussion section. The material covered in the results and discussion can, in turn, inform what information is essential to include in the introduction.  How many parents had experience with MDA programs for their school-aged children? Did those experiences influence their attitudes towards treatment for PSAC? | The study site description is placed in **2.2 Study area and setting,** because most information stated here was obtained from secondary sources and not necessarily during the field work, and it is common to describe the setting as part of the methodology.  Providing details for each of the 15 villages would extend the scope of this manuscript and may overload the reader.  In this section, schistosomiasis prevalence rates for the districts are provided as follows:  “In Bugiri, schistosomiasis prevalence in children between one and five years was measured at 35% in 2014 (12). A study at the shoreline of Lake Albert reported a prevalence of 46.5% among children aged five to ten years in 2016 (42).”  Recent prevalence rates on a village level are not available to us.  We have also already provided an overall description of the water and sanitation situation, economy, availability of services in the text.  We added the underlined words to one sentence, to inform the reader that there were also some private schools (beside the few public schools):  “In both study sites, there are few public primary schools, some private schools, no nearby secondary schools, and illiteracy among adults is common.”  We added the underlined sentence about the total amount of audio-recordings and average time of interview to **2.4. Data collection methods and interviews**:  “The interviews resulted in 97h24min of audio-recording, with an average time of 1h13min for individual interviews and 1h54min for FGDs.”  Knowledge is a major topic of this publication (**3.2.2 Parents’ knowledge about the signs/symptoms of schistosomiasis in children;** **3.2.3 Parents’ knowledge regarding schistosomiasis transmission modes in children; 3.2.4 Parents’ knowledge of preventive measures; 3.2.5 Parents’ knowledge about praziquantel for schistosomiasis treatment and mass drug administration),** yet, several publications already report on this topic. Therefore, we found it useful to investigate how knowledge is influenced, also in order to provide practical recommendations to policy and implementation officers, on how to best inform and sensitise parents. This manuscript underwent several drafting and re-structuring processes. Ultimately, we decided in favour of a holistic approach to provide the full picture instead of focussing only on the level of knowledge. The results provided are a careful selection, chosen to construct our argument and explain the influence of socio-cultural and structural barriers.  Experience with PZQ and MDAs is not the major focus of this article and will be part of a later paper, focussing on health seeking behaviour. |
| **Reviewer #2:**  This analysis highlights the main findings of the study and suggests areas for discussion and improvement, helping to strengthen the contribution of the article to the field of public health.   1. Awareness and Knowledge about Schistosomiasis   Comment: The results reveal a significant level of awareness about schistosomiasis among parents, which is encouraging. However, there are important gaps in knowledge regarding the transmission and prevention of the disease. The identification of erroneous beliefs, such as the association between  schistosomiasis and witchcraft underscores the need for more focused and culturally sensitive educational campaigns. I recommend that the authors discuss how these beliefs can be effectively addressed in future interventions.   2. Information Sources and Trust in VHTs    Comment: The identification of Village Health Teams (VHTs) as a primary source of information is a relevant finding. However, the limited training of VHTs regarding schistosomiasis suggests that they may not be fully equipped to provide accurate information. I suggest that the authors consider discussing strategies to improve the training of VHTs so that they can play a more effective role in disseminating health information. 3. Socio-Cultural and Structural Barriers    Comment: The analysis of barriers, such as high population mobility and illiteracy, provides valuable insight into the challenges faced in communicating health information. The identification of language barriers and limited trust in written information sources are critical issues that need to be addressed to ensure more effective information dissemination. I suggest that the authors discuss possible approaches to overcoming these barriers, such as using visual communication or oral narratives. 4. Information Dissemination and Campaign Impact   Comment: The results indicate that the dissemination of information about schistosomiasis has been fragmented and, in many cases, limited to logistical issues. The lack of comprehensive informational campaigns may be contributing to the persistence of knowledge gaps among parents. It would be helpful if the authors discussed the need for more structured and continuous health campaigns, especially those focused on hard-to-reach areas.   5. Acceptance and Use of Praziquantel   Comment: The acceptance of praziquantel as an effective treatment method is positive, but reports of side effects and the lack of information on how to deal with them are concerning. I recommend that the authors consider discussing the importance of providing complete information about side effects during MDA campaigns, which could improve acceptance and adherence to treatment.   6. Suggestions for Future Actions    Comment: Based on the results, future actions could include developing training programs for VHTs, creating more inclusive health campaigns, and implementing communication strategies that take into account the identified socio-cultural and structural barriers. I recommend that the authors consider exploring these suggestions in more detail, offering specific recommendations for future interventions. | Thank you for highlighting areas that are of policy and programmatic importance emerging from our study results. However, our study did not investigate interventions or strategies in detail, and we are limited in the conclusions we can draw from the research results. Some of our recommendations are already included in the text. Nevertheless, we have added some further points as determined to be appropriate into the discussion section.   1. In **4.1 Parents’ awareness and knowledge of schistosomiasis**, we discuss the diverse explanations of causes for schistosomiasis/kidadaa. The underlined words were added, for the terms “approach could lead” we deleted the word “could”:   “Continuous effort is needed to address the different perceptions of schistosomiasis transmission and to interact with treatment providers in Bugiri. Using the term “ekidada”, the term associated with witchcraft, to refer to schistosomiasis is not recommended, as this approach led to confusion in other settings (36).”  These are the recommendations that we can provide based on evidence from our data and the literature.   2. We added the underlined sentence to **4.2 Parents’ current**   **Information sources on schistosomiasis**:  “Our study also highlights the importance of VHTs and the challenges that they face to fulfil their tasks. Despite our finding that VHTs were parents’ primary information source and are trusted because they are part of the community, caregivers often expressed concerns about the limited knowledge of VHTs, leading to mistrust due to their lack of medical training. The Ministry of Health should look into ways to increase VHTs’ knowledge through improved and continuous training.”   3. We added the underlined sentence to **4.4 Socio-cultural and structural barriers to health campaigns:**   “The results of our study confirm these challenges and indicate that language barriers are a structural problem, as the government posts teachers and health professionals in public employment anywhere in Uganda, irrespective of their language skills. For the provision of information on schistosomiasis, this means that communication may be hampered but moreover, these experts may have a different cultural background and may have had little exposure to schistosomiasis beforehand and thus lack disease-related knowledge. Making use of visual communication material and reaching individuals through a peer-group approach could tackle this challenge.”   4. These are great suggestions but they are beyond the scope of our study. In **4. Discussion**, we already provided the following recommendations, drawing on the evidence from our data: - “Continuous effort is needed to address the different perceptions of schistosomiasis transmission and interact with treatment providers in Bugiri. Using the term “ekidada”, the term associated with witchcraft, to refer to schistosomiasis is not recommended, as this approach led to confusion in other settings (36).” - “These sometimes-guarded answers and the emotion expressed around participating in praziquantel campaigns indicate a need for a sensitive approach that recognises the sensitivity of the subject for some members of the community, and to take the time and effort to address their concerns.” - “Such findings show the importance of intense sensitisation of the recipient populations before introducing a new health intervention. Trainings for VHTs and community leaders are necessary to increase their knowledge, especially as they are important information providers for parents. It is also crucial to study and address existing fears and rumours effectively by developing targeted strategies to counteract them.” - “These findings indicate a gap in the availability and flow of information. We conclude that better health information infrastructure is needed, with a particular focus on parents and schistosomiasis information.” - “The Ministry of Health should look into ways to increase VHTs’ knowledge through improved and continuous training.” - “This underscores the need for accurate, comprehensive, and clear health information to avoid misunderstandings or misconceptions. Comprehensive information material that assists VHTs and community leaders with providing information to their communities could be helpful in keeping information correct.” - “With health professionals and the radio having the disadvantage of rarely being available in remote regions, we identified VHTs and the local council as the most accessible and accepted information distributors. Both require training to increase their knowledge of the disease and to educate them about the new medication, as well as facilitation with easily understandable messages and sensitisation material.” - “For the provision of information on schistosomiasis, this means that communication may be hampered but moreover, these experts may have a different cultural background and may have had little exposure to schistosomiasis beforehand and thus lack disease-related knowledge. Making use of visual communication material and reaching individuals through a peer-group approach could tackle this challenge.” - “This challenging situation sheds light on the strong attachment of parents in Hoima towards their VHTs. In contrast, parents in Bugiri had more hesitation against VHTs and were more in favour of receiving information about the new treatment from health professionals. These examples highlight the contextual differences among areas, requiring a sensitive balance between following standardised strategies and messages versus adapting health campaigns to the local context.” - “This finding could imply that government health campaigns are more likely to be accepted and effective in a stable, well-perceived economic and political environment. In cases where there is already a negative attitude towards government programmes, sensitisation could be more successful if carried out by more neutral parties such as health professionals or VHTs.”   5. This is a very important point and it is currently not addressed well enough by the Health Ministries, district authorities and relevant implementing health professionals and CHVs. We noted that different opinions on how much information shall be provided co-exist and it could be related to cultural sensitivities. We agree that providing more information on the effects of the drug would improve informed-decision making and might be a good way to avoid some of the rumours and take fears seriously. Certainly, there needs to be a balance what kind of information is provided, because we all know that the information leaflet of a medication contains also a lot of rare side events that might scare people from using the drug.   We added the underlined sentence to **4.1 Parents’ awareness and knowledge of schistosomiasis:**  “It is also crucial to study and address existing fears and rumours effectively by developing targeted strategies to counteract them. Providing more information on the effects of the drug could improve informed decision-making and demonstrate that authorities take community members’ fears seriously, thereby getting ahead of rumours that may otherwise circulate.”  6. Exploring these suggestions in detail are beyond the scope of the study and would not be evidence based. Nevertheless, in the conclusion, we do make suggestions for future studies and programmatic activities. Section **5. Conclusion** includes the following recommendations:  “Information campaigns that target caregivers of young children require thoughtful design and adaptation to local conditions to overcome socio-cultural and structural barriers. According to our substantial thematic analysis, there seems to be a preference that implementation needs to be carried out by trained and motivated VHTs and, at best, supported by health professionals and through the administrative structures of the local leadership. These are essential components to inform parents of PSAC so that they can make well-informed and trusted decisions for their children’s health. Although the results of in-depth, qualitative research are not intended to be widely generalisable, our findings indicate the importance of considering contextual factors such as language barriers, level of literacy, population movements, rumours and trust in government programmes for planning the (pilot) roll-out of the schistosomiasis treatment for PSAC. By using contextualised approaches, local barriers can be overcome.” |
| **Reviewer #3:** This is overall a clearly written paper articulating a set of important findings, well situated within relevant and supportive literature. The descriptions and use of photographs in the methods give the reader a strong sense of what the communities are like, presented succinctly. There is a really nice summary of the challenges on lines 486-493: these show how geographical location, education levels, and technology intersect. This is very clear, and it is good to see this coupled with expansion of content in Table 3. The findings reinforce the findings of other studies, but do an excellent job of why attention to wider context is important.  My main critique (if critique is even a useful word here) is about the way that local ways of knowing are presented in the paper and the ethical implications of this for producing meaningful change. The below (and I apologise that it is so long) is intended as an invitation for reflection on how the language we use can both help and hinder efforts to produce meaningful change. They are an invitation to build on the important strengths of this paper only.  The first of these is your distinction between an ‘IDI’ (in-depth interview) and ‘KII’ (presumably key informant interview). In-depth is a description of a methodological approach – that the interview seeks to achieve depth – and is an approach that can be taken with any interviewee. KII suggests that some people are more ‘key’ than others – and thus that those others (here, presumably community members, who are the experts in their own lives) are less, although most of the findings focus on their interviews. Is this a distinction you want to reinforce? Interestingly, in your presentation of findings, you do not reinforce, but indeed blur, it. For example, ‘A 48 year old other from Bugiri…’ quoted with ‘KII, mother, Bugiri (501)’ as the descriptor after the quote, or ‘KII, mother, Hamia (209)’ earlier on. Given that in your analysis there does not seem to be a meaningful distinction between KII and parental interviews, why set up this distinction at al l? Or, alternatively reflect on what it means that at least some of your ‘key’ informants answered as parents? What does this tell you?  I further invite you to reflect on the implications of describing a group or individual’s way of understanding how transmission works as ‘a lack of understanding’ (352), a ‘misconception’ (379, 480), ‘belief’, ‘misbelief’ or ‘false assumptions’ (408).  Think here about how you have characterised Parker et al (2011) in your discussion of the literature (151-154): you draw on this text to argue ‘local understandings of NTDs did not mirror biomedical knowledge’. Yet, in the cited paper, Parker et al do not argue that ‘local understandings of NTDs did not mirror biomedical knowledge’ but speak of ‘divergence between local and biomedical understandings’. This is a subtle but powerfully important difference – biomedical and local frames of understanding are different rather than one being false or lacking.  When we as scholars and scientists speak to people to understand their world, and then articulate that to others what does it mean to describe those understandings as false, misconceptions, or lacking? I therefore advise you to look again at Parker et al 2011 and note the way they use language. You may also find Farmer’s 1990 ‘Sending Sickness’ useful – this is an excellent example of how multiple understandings of the cause of a sickness can circulate within a single community. If we want to make change through understanding, can we do that while calling our interlocutors’ understandings wrong?  Related to this I notice is “bewitch” in inverted commas. Again, I invite you to reflect on the ethical implications of this punctuation choice: it implies that you do not take seriously bewitchment as a transmission mechanism. There is an argument in anthropology that reality is that which produces effects in the world, and bewitchment as a means of transmission (whether we or anyone else believes that bewitching is ‘real’ or not) produces effects in the world (again, Farmer’s ‘Sending Sickness’ and also AIDS and Accusation illustrate this beautifully). People seek out particular kinds of treatment (and perhaps not others) based on what they understand to be the cause. The preventative methods proposed for avoiding kidaada in the paper are entirely consistent with understandings of how kidaada is caused as presented in the paper. There are thus no ‘misconceptions’ (411) about prevention here – what there is, however, is a difference between how different groups ( ie you and the community you seek to describe) understand schistosomiasis is transmitted, how kidaada is transmitted and the relationship between schistosomiasis and kidaada. What are the implications, then, of taking seriously the frames applied by the communities we wish to work with?  Consequently, I was fascinated that the solution around lines 808-810 does not include engaging with and taking seriously witchcraft. If at least 1/3 people use it as a form of explaining causality and meaning-making, why not engage with it and take it seriously? How can you push your recommendations beyond simply ‘recognis[ing] the sensitivity of the subject’? Do we want to work with localised forms of sense-making or seek to replace them? What are the ethical implications of (either) answer to this question?  For this, it may be worth thinking about how you communicate some of the complexity around trying to talk about a singular thing (‘schistosomiasis’, ‘bilharzia’) when ‘it’ clusters around different kinds of overlapping symptoms and causes. You do this beautifully in this paper. Your work here is important and makes visible the way in which what schistosomiasis is in these communities (and between these communities and research communities) is not simple or consistent. This is a real strength of the paper, as this complexity is hard to communicate and you have done so succinctly and effectively. I invite you to build on this strength in relation to the above.  I was particularly struck by your presentation of participants who did not use PZQ. Resistance to PZQ in themselves seems important for understanding (barriers to uptake) in a paediatric formulation. You cite one participant who was clearly uncomfortable about telling you why they did not take it in the results and go on to say ‘Community members with different opinions may not always have opened up about them in interviews’ (474): did any community members tell you why they did not use PZQ? If not, what does reluctance to say why not tell you? Did people discuss making different choices about PZQ use for themselves or their children (ie refuse it themselves, but support their children taking it or vice versa)? I thus recommend that if there were any participants who did tell you why they did not take PZQ that you include an analysis of those people’s stated reasons; if they did not, and people who told you did not could or would not tell you why, keep this important point in, and reflect on it in the discussion. You can push your analysis beyond ‘shame’ (799) here. None of the material presented supports the idea that it is ‘shame’ that drove their non-disclosure, however what that quote does show is that when pressed some people who don’t take PZQ they don’t want to tell you why. Given the ways in which explanations for non-use are presented as something that others do in the rumours section, are there other ways of making sense of this than individualised feelings of shame? Great note of socially desirable responding though. Building on strengths in the paper, the paragraph on reasons why other people refuse PZQ/other vaccines blurs into the paragraph on impacts of new fishing regulation and enforcement. This very clearly shows how choices made about medication are not made in isolation from wider relations of power and larger political and economic forces. This is excellent.  Related to this this, the discussion on rumours is very important. This really adds to the paper. However, in light of the invitation to explore more culturally relativist perspectives above, is it worth considering how what you have described as ‘rumours’ could also be understood as articulations of people’s fears? A ‘rumours’ framing has implicit within it that if only people were provided with the ‘facts’, different choices would be made. However, framing this through fears opens up different forms of engagement that start from and take seriously individuals’ and communities’ own understandings of the world. None of the above are recommendation but rather an invitation to pay attention to how the language we use can reinforce or challenge hierarchical knowledge-relations, and create space to situate our responses and solutions in ways that work with the worlds our research participants inhabit.  Figure 5 (ln 773) captures most but not all of the material covered in the paper: there’s an important, fundamental mistrust of VHTs’ low level of training/community insider identity in the paper that is important, but not quite captured by ‘trust or mistrust in certain professions and political parties’. I also wonder in what ways ‘level of education’ is a barrier to ‘accessing and understanding accurate health information’ – is literacy/numeracy required for this? Is the burden on those seeking to sensitise perhaps to adapt their tools the community, rather than a limitation of the community?  You have not evidenced how ‘customs and habits’ function as a barrier in the paper, but more importantly than that, I invite you to consider the ethical implications of situating responsibility for intervention failure on the ‘customs and habits’ of the target population. Pot’s 2019 paper ‘INGO Behavior Change Projects: Culturalism and Teenage Pregnancies in Malaw i’ conveys Fassin’s concept of ‘culturalism’ really clearly and I advise you read it and consider the ethical implications of presenting the culture of the target population as a barrier. | Thank you for this reflection which has indeed served as an invitation to explore these ideas further and to better represent the analysis and the experiences and perceptions of research participants.  Thank you for catching the inconsistency use of the terms IDI and KII when referring to parents. We discussed the definitions of IDIs and KIIs again and decided to call all interviews IDIs in this manuscript. Thus, all previous terms “key-informant interview” were integrated within with the term “in-depth interview” and all abbreviations “KII” were replaced with “IDI”.  Changes were made in the **abstract**, **method** section including table 2 and **results** (quotes). In addition, the word “key” was deleted when referring to informants (~~key~~ informants).  Thank you a lot for pointing out the problematic use of these terms (“misconceptions”, “lack of understanding”,..) here and later also the term “rumour”. We appreciate these comments and also agree, that the term rumour should be used more critically. Most of the time, “rumours” have now been changed in the manuscript to “fears”. A few times, both terms are used. We went through the manuscript and tried to reformulate them as follows:  **Abstract:**  Despite high parental awareness of the disease and knowledge of signs and symptoms, we identified less familiarity with its transmission and prevention. With limited communication channels, VHTs emerged as the primary information sources but reflected varied local understandings of schistosomiasis.  …  Other mentioned socio-cultural and structural barriers to information flow included language barriers, illiteracy, high population movement, fears, and resentment from restrictive government fishing laws.  **Author summary:**  We identified several fears, for example that mass treatments could be a hidden population reduction strategy.   1. **Introduction**   We added “and believes” to the following sentence:  “Similarly, adults’ knowledge and beliefs will influence how children are taught and told to behave and act, for example, to avoid playing and swimming in lakes and not defecate in the open. However, changing WASH practices requires much more than raising knowledge levels – at the minimum, adequate water and sanitation must be available for daily needs.”  We changed the following sentence:  “Several studies in SSA and also in Uganda have reported on the existence of local explanatory models surrounding schistosomiasis – often labelled as “misinformation” and “misconceptions”(3, 28, 30, 31).”  We changed the terminology:  “A study in Nebbi (now Pakwach) district at Lake Albert, Uganda, has shown that local understandings of neglected tropical diseases (NTDs) diverged from biomedical understandings.”  **3.2 Existing awareness and knowledge of schistosomiasis**  The following sentence was changed: **“**Parental knowledge concerning signs and symptoms, the transmission, and preventive measures of schistosomiasis diverged from biomedical knowledge.”  **3.2.3 Parents’ knowledge regarding schistosomiasis transmission modes in children**  We exchanged the word “was” into “remained” in the following sentence:  “Few parents were aware that worms are part of the transmission cycle, and amongst those, their knowledge remained limited to simply knowing that worms are part of the process.”  The following sentence below a quote was rephrased, avoiding the term “lack of knowledge”:  “You see, when people go into the water, they find those parasites that can give you bilharzia. Others drink non-boiled water, then they later complain about stomach-aches, yet they drink dirty water. That is a problem. Some people defecate in the water while they are fishing, and other people come and fetch that same water. All this contaminates the water. – KII, mother, Hoima (209)  This quote implies knowledge about open defecation and its relation to schistosomiasis, even though schistosomiasis is not transmitted through ingesting contaminated water.”  We replaced the term “misconception” with “assumption”: The assumption that drinking contaminated water from the lakes causes schistosomiasis was common among parents.  We have deleted the inverted commas around the term bewitch and change the sentence as follows: “In this belief, a person can bewitch another person to be affected by kidaada by cursing or poisoning their food – mostly said to be done by neighbours.”  **3.2.4 Parents’ knowledge of preventive measures**  We changed “false assumptions” into “different ideas”:  “Just as with disease transmission, there were also different ideas about infection prevention.”  We changed the wording:  “Some parents from Bugiri, who believed that witchcraft causes schistosomiasis/kidaada consequently stated other measures to prevent the disease:”  In section **3.2.5 Parents’ knowledge of preventive measures,** we state the following and added the underlined sentences:  “In the IDIs and FGDs with parents, only four participants explicitly mentioned not taking praziquantel themselves: one of them was a mother living with two children in a very hard-to-reach area: [Quote]  The last quote indicates that not taking the medication can be taboo, and some respondents did not feel comfortable speaking about it. Another father of seven children, who chose not to allow his children to take praziquantel, shared during a FGD his reasoning resulting from a previous negative experience:  R8: An example is me, I also don’t allow my children to take drugs anyhow because I had a bad experience. It was around 2017 when my child was given praziquantel at the age of 7 years. The drug treated my child terribly in that I had to spend a lot of money to treat the child. If I had a choice, I would not allow them to take that drug because I end up spending a lot of money treating the side effects. My child collapsed under the sun after being given the drug. When I took my child to the hospital in [location X] we were referred to Hoima referral hospital. Upon reaching the hospital I was told that my child was given an overdose. I spent 450,000 shillings to treat my child. That is why I can never allow my child to take drugs again. – FGD, father, Hoima (008)  Decisions surrounding reluctance to treat their children with praziquantel are born out of personal experiences.”  **3.3 Available, trusted and desired information sources about schistosomiasis in children**  We changed the wording: **“**After identifying local ideas and explanations among parents on transmission routes and preventive measures, that diverged from biomedical explanations, we subsequently studied how parents received information about the diseases, especially among children and asked through which channels they would like to be informed about the new paediatric praziquantel.”  Added “would have” to increase clarity: “Parents in these areas would have preferred verbal communication through radio, TV, or community leaders, but the availability of TV and radio posed challenges.”  **Table 3**: We changed the following statements:  “Quality of information provided dependent on knowledge of the parents’ information source: lower information quality provided by VHTs and community leaders, higher quality provided by teachers and health professionals”  “Fears concerning praziquantel”  “Rumours about the “real” aim of the treatment and fears concerning praziquantel can affect acceptance 🡪 Consider rumours and fears during the development of sensitisation campaigns”  **3.5.1 Knowledge among providers of information to parents**  We changed the terminology in the following sentences:  “Two individuals from the group of community leaders, a religious healer and one teacher (who had moved recently from a different region), could not name any symptoms.”  “In general, knowledge of disease transmission through water contact was modest.”  “Few knew that the disease life cycle involves snails, while others knew that snails are involved. It became clear that local explanations for schistosomiasis transmission among the communities – to which the VHTs and community-leading figures belong – were widespread and that these informants also played a role in parents’ level of knowledge.”  We changed the terminology of the title of section **3.6.3 Fears of praziquantel and mistrust in governmental health campaigns** (previously 3.6.3 Rumours of praziquantel…) and also change the term “rumour” into “fear”, added the words “fears and”, and changed the word “claims” into “narratives” in the following sentences:  “Fears surrounding praziquantel, suspicions about the government’s true intention behind health campaigns, negative experiences with government policies, and rejection of the ruling political party were identified as key themes contributing to fear, mistrust, and the undermining of health promotion campaign effectiveness.  During the interviews and discussions, we noted that several fears and rumours concerning schistosomiasis and its treatment were spread among parents in the targeted communities. The rumours ranged from narratives that VHTs and health professionals were stealing schistosomiasis drugs from MDAs to sell them privately, to fears that the drugs would be part of a hidden family planning agenda (or perceived worse scenarios), ultimately harming or even killing children. In Bugiri, we also heard that parents associate strong (side) effects of the drugs with potential overdosing.”  “One health professional in Bugiri referred to the lake as a “toilet of ours”, reflecting that it is an often used place for defecation, and is linked to the scarcity or absence of latrines.”  “These findings underscore the influence of fears and rumours, the current political climate, and the ensuing sentiments on shaping perceptions of other government programs, including public health campaigns.”  **4.1 Parents’ awareness and knowledge of schistosomiasis**  We changed the following sentences, newly included text is underlined:  “Despite their awareness, parents’ knowledge of schistosomiasis in PSAC varied and we detected modest biomedical knowledge concerning prevention and transmission.”  “We also detected other local ideas and explanations regarding schistosomiasis causes and preventive measures. In Bugiri, the belief that someone can bewitch or curse a neighbour and cause them to suffer from a schistosomiasis-like disease called kidaada persisted amongst approximately one-third of the respondents.”  “If community members have different understandings of transmission, such as the belief in witchcraft, it may hinder treatment efforts. Continuous effort is needed to address the different perceptions of schistosomiasis transmission and interact with treatment providers in Bugiri.”  In **4.1 Parents’ awareness and knowledge of schistosomiasis**, the underlined words were changed and the term “shame” is not used anymore:  “Concerning knowledge about using praziquantel as preventive therapy, we noted that participants sometimes exhibited discomfort when disclosing that they did not take the medicine during MDAs, and some avoided talking about it.”  We replaced the word “rumours” with “fears”:  “We identified fears among participants that health campaigns could be part of a hidden governmental family planning agenda or even plans to reduce the population. Several studies identified fears that praziquantel may affect fertility (34, 36, 51).”  We changed the terminology in the following sentence:  “Trainings for VHTs and community leaders are necessary to enable them to inform parents sufficiently. It is also crucial to address existing fears and rumours effectively by developing targeted strategies to counteract them.”  **4.2. Parents’ current information sources on schistosomiasis**  We replaced the words “misunderstandings and misconceptions” with the underlined words:  “This underscores the need for accurate, comprehensive, and clear health information to avoid knowledge discontinuity.”  We added your beautiful conclusion to section **4.4 Socio-cultural and structural barriers to health campaigns** as underlined:  “This example shows that fear and rejection of some political activities can or may also lead to the rejection of other initiatives if they are perceived as political, which could result in rejecting a new health campaign, and how choices made about medication are not made in isolation from wider relations of power and larger political and economic forces. The significance of underlying political and economic situations was also pointed out by other researchers.”  We replaced the word “low” with “modest”:  “They concluded from a comparison of different sites in Uganda that the success of NTD control in one district (Busia) was promoted by the positive political and economic situation of this district because the knowledge level of this population was as modest as that of the compared districts, that had less success with MDAs (34).”  Thank you for your recommendations and for providing this insightful literature. We adjusted **Figure 5**: added the underlined words “fears and rumours”, deleted the box stating “customs and habits”.  Concerning the comment about the “level of education” as barrier to assessing information: in case of illiteracy, written material cannot be assessed, but we already stated literacy in another box. Therefore, we deleted the “level of education”-box and replaced it by dividing the previous box “Trust or mistrust in certain professions and political parties” into two separate boxes named “Confidence in VHTs’ knowledge” and “Trust or mistrust in certain political parties”.    However, the appropriateness of training and information material is an important point! The materials and campaigns need to be better tailored to the target populations, e.g. by using visuals as we pointed out in the discussion. |
| Smaller points:  • In line 599-600 you state that ‘it became clear that misbeliefs by VHTs and other community-leading figures contribute to parents’ false assumptions’. Given you state above that with the exception of teachers (who demonstrated a high level of knowledge in line with MoH/biomedical understandings), the same range of understandings and assumptions is shared across parents, community leaders and VHTs, can you evidence from your material that it is specifically causal and not simply a reflection of the wide range of ideas about symptoms, causes etc present in the community? If not, soften your language.  • Be careful of extrapolating out from a single participant to make claims about culture: Can you use one person referring to the lake as a ‘toilet of ours’ to make a claim that this is a ‘culturally accepted’ place to defecate? Are there latrines? Are they clean? Are they affordable? What did you find in your observations? Is it only ‘culture’ that drives people to defecate at the lake? Can you evidence this? If not, be very careful about making claims about ‘culture’ as a driver of practice (again, Pot 2019 will likely be useful here).    • The discussion of lack of trust in VHTs is a really important aspect of the paper: there is real subtlety of analysis here. There is thus an opportunity on line 634 to build on this: low literacy rates, restricted access to info, and ‘relying on the scarce information from VHTs’ does not capture the lack of trust in VHTs articulated earlier. Just a few additional words here would make a huge difference.  • Could you build on ‘Training for VHTs and community leaders are necessary to increase their knowledge, especially as they are important information providers for parents’ (819-820)? The paper really beautifully shows that local community perceptions about VHTs as no more educated than everyone else etc really impacts on the ways their messages are received. You’ve shown really nicely that community leaders have powerful influence, even if political figure do not. Therefore can you make more of this here?  • Finally, could the fact that even with knowledge, practices cannot necessarily change due to the material conditions of people’s lives be moved up?  This fact – and the reality of witchcraft as a meaningful force in the world for a significant proportion of your participants – might be important framing for subsequent recommendations about sensitisation efforts. | - We changed the sentence into: It became clear that misbeliefs among the communities – to which the VHTs and community-leading figures belong – were widespread and that these informants also play a role in the extend of parents’ knowledge.   **3.5.1. Knowledge among providers of information to parents**  We changed the underlined words, deleting the reference to culture:  “One health professional in Bugiri referred to the lake as a “toilet of ours”, reflecting that it is an often used place for defecation and is linked to the scarcity or absence of latrines.”   - In **3.6 Socio-cultural and structural barriers to schistosomiasis communication,** we added the underlined words to capture the trust issue:   “Low literacy rates and restricted access to official information sources, relying on the scarce information from VHTs, whom some parents did not trust, and health professionals, further complicated by language barriers and a high population movement, were challenges for accessing schistosomiasis information, sustainable health education, communication and subsequent health literacy.”   - The recommendation to provide and improve continuous training for VHTs is an important result of our study.   We added to 4.2. Parents’ current information sources the sentence:  “The Ministry of Health should look into ways to increase VHTs’ knowledge through improved and continuous training.”  Related to this, we would also like to point out the recommendation “Comprehensive information material that assists VHTs and community leaders with providing information to their communities could be helpful in keeping information correct” at the end of this section. In addition, this point is taken up in the following section **4.3. Parents’ preferred sources of information for the new paediatric treatment:**  “With health professionals and the radio having the disadvantage of rarely being available in remote regions, we identified VHTs and the local council as the most accessible and accepted information distributors. Both require training to increase their knowledge of the disease and to educate them about the new medication, as well as facilitation with easily understandable messages and sensitisation material.”   - We added text about latrines and community toilets to **4.1 Parents’ awareness and knowledge of schistosomiasis**:   “The situation concerning toilets and latrines is very similar. In one village, the houses are built so closely together leaving no room for toilets. Some of the latrines they dug themselves are too shallow, resulting in faeces being flushed into the lake during heavy rains and flooding. There were communal toilets, but they were either unlocked and not very clean, or locked requiring request and retrieval of the key from someone. In view of the unsatisfactory sanitary infrastructure, it is unlikely that open defecation will decrease, despite knowledge about schistosomiasis transmission.”  We highlighted the reality of witchcraft in the manuscript, however we have not identified the most effective way, how to consider it as part of the sensitisation, also because the different explanatory models for schistosomiasis vary so much. It would require more research (scoping review how witchcraft is considered in other countries / related to other diseases) and piloting of methods.  Based on your wording, we added the underlined sentence to **4.1. Parents’ awareness and knowledge of schistosomiasis**  “Continuous effort is needed to address the different perceptions of schistosomiasis transmission and to interact with treatment providers in Bugiri. The reality of witchcraft as a meaningful force in the world for a significant proportion of our participants would be important to take into consideration for future sensation efforts. Using the term “ekidada”, the term associated with witchcraft, to refer to schistosomiasis is not recommended, as this approach led to confusion in other settings (36).” |
| **SUMMARY AND GENERAL COMMENTS** |  |
| **Reviewer #1**: In this study, the authors describe an impressive qualitative investigation involving parents, health workers and community leaders in two schistosomiasis-endemic districts in Uganda. Their study anticipates an important event in schistosomiasis control, as the Ugandan government prepares to pilot the distribution of a newly approved pediatric formulation of the anti-helminthic drug praziquantel. The study aims to capture knowledge of schistosomiasis, its treatment and sources of information in this population. Overall, this paper merits publication but there are several shortcomings in the current version that will require major revision before it is acceptable for publication.  In addition to the methods and results comments above, I have outlined requests below for additional background and contextual information:  It is never mentioned which schistosome species affect the study area.  Similarly, the authors do not describe the basic details of the transmission cycle, despite it being a topic discussed in interviews.  What does a “positive opinion” from the European Medicine Agency mean? What have clinical trials of arpraziquantel shown?  What is the Ugandan government’s timeline for piloting drug distribution in the pediatric population? Incorporating answers to these questions in the Introduction section is important contextual information for the current study.  How do MDA programs tend to work in Uganda? My general understanding is that these programs tend to be school-based to reach school-aged children. After noting in line 221 that there are few public primary schools in the study sites, though, I’m wondering if that assumption is incorrect. | Thank you for taking the time to review our manuscript. We appreciate your valuable recommendations and hope this revised version sufficiently addresses the identified limitations.  We added the requested information to **2.2 Study area and setting**:  “In Uganda, Schistosoma *mansoni* is the predominant species responsible for causing intestinal schistosomiasis (13).”  We added a description of the transmission cycle to **1 Introduction**:  “People or animals infected with schistosomes transmit schistosomiasis through contamination of freshwater with faeces or urine containing parasite eggs. Those eggs hatch in the water and release immature larvae, which penetrate snails where they undergo further larval development. People become infected when larvae, that are released from freshwater snails, penetrate the skin during contact with the contaminated water. In the body, these larvae mature into adult worms and live in blood vessels around the bladder or the intestine, where females lay eggs after mating. Some eggs exit the body through faeces or urine, continuing the life cycle of schistosomiasis, while others become lodged in tissues, triggering immune responses that can lead to chronic inflammations and subsequent organ damage (1).”  We added the reference about the results of the clinical trial and added the underlined half-sentence about the trial results:  “It has positive attributes such as being smaller in tablet size and dose, dissolvable in water and improved in taste and was found in a clinical trial to be safe and effective (22, 23)”.  We added the underlined half-sentence to **1 Introduction:**  “In preparation for the (pilot) roll-out of the novel paediatric praziquantel formulation for PSAC within the ADOPT-programme, which is planned for the end of 2024, we conducted a qualitative study to support the adoption of arpraziquantel in routine health care for PSAC in Côte d’Ivoire, Kenya (37, 38), and Uganda.”  We added a sentence to explain MDA programmes into **2.2 Study area and setting**:  “Besides the distribution of praziquantel at schools, the remaining population (older than five years) receives the medication from health professionals or VHTs, either at fixed points in their village or through a door-to-door approach directly at their homes.” |
| Reviewer #3: This review is, I appreciate, a lot. And I hope you are able to take it in the spirit that it is intended: which is that this is an important paper, with moments of wonderful subtlety, complexity and compassion. It tells an important story and makes an important argument well. The above is thus offered in the spirit of taking something very good and making it excellent – or at the very least, inviting you to reflect on the value of starting from the worldviews of the communities with which we work, rather than seeking to supplant them (which I am not sure is your intention, just perhaps an accidental by-product of language choices). You do such a good job of capturing the ways in which seemingly unconnected political events (eg changes in fishing regulation or teacher recruitment practices) profoundly shape and inform understandings, I wish merely to invite you to build on these excellent strengths by pushing yourselves to reflect on language and framings a little more. Overall, I have found this paper insightful and subtle and hope to see it in print soon. | Thank you for taking the time to review the paper and provide these thoughtful recommendations, which helped us to reflect further and to add and change important components, thereby improving the manuscript. We have addressed your comments and hope that you will find the manuscript improved. |
|  |  |
| OTHER CORRECTIONS | Additional references:  “23. N'Goran EK, Odiere MR, Assandé Aka R, Ouattara M, Aka NAD, Ogutu B, et al. Efficacy, safety, and palatability of arpraziquantel (L-praziquantel) orodispersible tablets in children aged 3 months to 6 years infected with *Schistosoma* in Côte d'Ivoire and Kenya: an open-label, partly randomised, phase 3 trial. The Lancet Infectious Diseases. 2023;23(7):867-76.  40. Republic of Uganda. Bugiri District. Local government n.d. [29.10.2024]. Available from: <https://bugiri.go.ug/lg/location-size>.  41. Republic of Uganda. Hoima District. Local government n.d. [29.10.2024]. Available from: <https://hoima.go.ug/population/>.” |
|  | **List of authors’ affiliations:** We updated the affiliation for Nr 1, corrected one letter of Nr 7 and corrected the alphabetical order for the names in the middle.  We corrected the number of total interviews and involved individuals in table 1 and text in **2. Methods** and also in the **Abstract.** Previously, an interview from Hoima was incorrectly counted twice (previously 62 IDIs, now 61, previously 142 individuals, now 141).  We corrected the reference number for study registration at clinicaltrials.gov in the section **2.1 Ethical considerations:**  “The study was registered at clinicaltrials.gov (ID NCT05350462).”  Corrected a few verbs from present tense into past tense in results sections.  **2.3 Study design, population and recruitment**: Deleted “levels of” in the following sentence: “This study utilised a cross-sectional qualitative design to assess parents’ knowledge, preferred information sources and socio-cultural and structural barriers for communicating schistosomiasis information.  **2.4 Data collection methods and instruments:** We deleted the underlined part “parents/guardians (later only referred to as “parents”) from the following sentence, because this was already introduced before in **2.3 Study design, population and recruitment**.  “Ten FGDs were conducted: four with parents of PSAC and one with VHTs in each of the two districts (**Fehler! Verweisquelle konnte nicht gefunden werden.**)”.  **2.5. Data analysis:** We deleted the word “the” between the underlined words. “Based on the research questions and the experiences during fieldwork, the researchers developed a thematic coding tree to analyse the qualitative data.”  We changed the word “section” and replaced it with “and analysis” in the following sentence:  “The analysis was discussed between the TUM and MU researchers. Later, validation meetings were held in each district to disseminate study findings and gather feedback. The feedback that was received was used to refine the results and analysis.”  **3.2. Existing awareness and knowledge of schistosomiasis:**  Changed previous sentence into:  “Parental knowledge concerning signs and symptoms, the transmission, and preventive measures of schistosomiasis diverged from biomedical knowledge.”  **3.2.3 Parents’ knowledge regarding schistosomiasis transmission modes in children:** added underlined words:  “In this belief, a person can bewitch another person to be affected by kidaada by cursing or poisoning their food – mostly said to be done by neighbours.”  **3.3 Available, trusted and desired information sources about schistosomiasis in children:** changed wording of the sentence:  “After identifying local ideas and explanations among parents on transmission routes and preventive measures, that diverged from biomedical explanations, we subsequently studied how parents received information about the diseases, especially among children and asked through which channels they would like to be informed about the new paediatric praziquantel.”  **3.3.2 Health professionals as information sources**  Added word “the“  “The services at the only Health Centre III (higher level health centre), serving the study villages in Hoima, were disrupted due to the 2020 floods.”  **3.5.2 Content of information provided to parents**  We deleted the brackets around the last sub-sentence:  “Concerning MDAs, parents were mainly informed by VHTs at community meetings about the logistics (place and time) and were advised to eat before taking the drug to reduce nausea and other side effects.”  **4.1** **Parents’ awareness and knowledge of schistosomiasis**  Changed terms of this sentence into:  Trainings for VHTs and community leaders are necessary to enable them to inform parents sufficiently.  Deleted “study and“ in the following sentence:  “It is also crucial to address existing fears and rumours effectively by developing targeted strategies to counteract them.” |
